# Topological reinforcement as a principle of modularity emergence in brain networks

**DOI:** 10.1162/netn_a_00085

**Published:** 2019-05-01

**Authors:** Fabrizio Damicelli, Claus C. Hilgetag, Marc-Thorsten Hütt, Arnaud Messé

**Affiliations:** Institute of Computational Neuroscience, University Medical Center Eppendorf, Hamburg University, Hamburg, Germany; Institute of Computational Neuroscience, University Medical Center Eppendorf, Hamburg University, Hamburg, Germany; Department of Health Sciences, Boston University, Boston, Massachusetts, United States of America; Department of Life Sciences and Chemistry, Jacobs University, Bremen, Germany; Institute of Computational Neuroscience, University Medical Center Eppendorf, Hamburg University, Hamburg, Germany

**Keywords:** Modularity emergence, Neuronal plasticity, Topological reinforcement

## Abstract

Modularity is a ubiquitous topological feature of structural brain networks at various scales. Although a variety of potential mechanisms have been proposed, the fundamental principles by which modularity emerges in neural networks remain elusive. We tackle this question with a plasticity model of neural networks derived from a purely topological perspective. Our topological reinforcement model acts enhancing the topological overlap between nodes, that is, iteratively allowing connections between non-neighbor nodes with high neighborhood similarity. This rule reliably evolves synthetic random networks toward a modular architecture. Such final modular structure reflects initial “proto-modules,” thus allowing to predict the modules of the evolved graph. Subsequently, we show that this topological selection principle might be biologically implemented as a Hebbian rule. Concretely, we explore a simple model of excitable dynamics, where the plasticity rule acts based on the functional connectivity (co-activations) between nodes. Results produced by the activity-based model are consistent with the ones from the purely topological rule in terms of the final network configuration and modules composition. Our findings suggest that the selective reinforcement of topological overlap may be a fundamental mechanism contributing to modularity emergence in brain networks.

## Author Summary

The self-organization of modular structure in brain networks is mechanistically poorly understood. We propose a simple plasticity model based on a fundamental principle, topological reinforcement, which promotes connections between nodes with high neighborhood similarity. Starting from a random network, this mechanism systematically promotes the emergence of modular architecture by enhancing initial weak proto-modules. Furthermore, we show that this topological selection principle can also be implemented in biological neural networks through a Hebbian plasticity rule, where what “fires together, wires together” and, under proper conditions, the results are consistent between both scenarios. We propose the topological reinforcement as a principle contributing to the emergence of modular structure in brain networks. This addresses the gap between previous pure generative and activity-based models of modularity emergence in brain networks, offering a common underlying principle at the topological level.

## INTRODUCTION

Modularity, the presence of clusters of elements that are more densely connected with each other than with the rest of the network, is a ubiquitous topological feature of complex networks and, in particular, structural brain networks at various scales of organization (Sporns & Betzel, 2016).

Modularity was among the first topological features of complex networks to be associated with a systematic impact on dynamical network processes. Random walks get trapped in modules (Rosvall & Bergstrom, 2008), the synchronization of coupled oscillators over time maps out the modular organization of a graph (Arenas, Díaz-Guilera, & Pérez-Vicente, 2006), and co-activation patterns of excitable dynamics tend to reflect the graph’s modular organization (Messé, Hütt, & Hilgetag, 2018; Müller-Linow, Hilgetag, & Hütt, 2008; Zhou, Zemanová, Zamora, Hilgetag, & Kurths, 2006). At an abstract level, modularity in the brain is thought to be important for information processing, the balance segregation and integration, as well as system evolvability in the long temporal scale, among others (Sporns & Betzel, 2016). More concretely, the modular organization of brain networks forms the substrate of functional specialization (e.g., sensory systems; Hilgetag, Burns, O’Neill, Scannell, & Young, 2000), contributes to the generation and maintenance of dynamical regimes (e.g., sustained activity; Kaiser & Hilgetag, 2010) and criticality (Wang & Zhou, 2012), and supports the development of executive functions (Baum et al., 2017). Thus, modularity is a key component of structural brain networks with important functional consequences.

Although a number of potential mechanisms have been proposed for the creation of modules (Ellefsen, Mouret, & Clune, 2015; Gómez-Robles, Hopkins, & Sherwood, 2014; Henderson & Robinson, 2013), the fundamental generative principles of the emergence of brain modules remain elusive, both algorithmically, in terms of the necessary topological changes for generating them, as well as with respect to a plausible biological implementation, that is, the realization of such topological changes through physiological mechanisms.

Generative models constitute a common approach to the study of the formation of global patterns of brain connectivity (Betzel & Bassett, 2017), where, broadly speaking, networks are allowed to grow in size and/or density according to specific rules. These models might be either based on fundamental concepts, such as developmental time windows (Kaiser & Hilgetag, 2007) and nonlinear growth (Bauer & Kaiser, 2017), constrained by experimental criteria, for instance, including geometric and topological features found in empirical connectivity data (Betzel et al., 2016), or based on dynamical factors, such as synchronization between nodes (Gong & van Leeuwen, 2003). Given the well accepted role of synaptic plasticity in brain development and activity-dependent adaptation (Abbott & Nelson, 2000), other perspectives focus on changes driven by such local plasticity mechanisms in physiologically more realistic models. A considerable proportion of this work aims at explaining empirically observed distributions of physiological parameters at the cellular scale, such as synaptic weights (Effenberger, Jost, & Levina, 2015), and only a few studies have paid attention to topological aspects, such as the proportion of local motifs (Stone & Tesche, 2013). Some of the mentioned modeling studies showed an emergence of modular network structure and attempted to provide an underlying mechanism based on the reinforcement of paths between highly correlated nodes (Jarman, Steur, Trengove, Tyukin, & van Leeuwen, 2017). Yet, the problem of a topological developmental gradient, along which network changes should occur during the rewiring process in order to promote the emergence of modules, was not explicitly investigated.

Addressing this challenge, we propose a model bridging the gap between purely generative models (e.g., “homophily-driven models”) and activity-based models (e.g., Hebbian-like plasticity models), with a binding element at the topological level. That means, we formulate a model that focuses on the contributions of pure topological changes, being different from previous models because it is agnostic with regard to the dynamical regime representing neuronal activity. At the same time, the presented model attempts to go a step further beyond the above mentioned generative modeling work, because it can be instantiated in a biologically more realistic fashion than such models, since they rather describe the end result of the network configuration and do not focus on the actual mechanistic explanation.

Concretely, the present study proposes a generative principle of structural modular networks through topological reinforcement (TR). This rewiring rule, derived from a purely topological perspective, constitutes a plausible underlying mechanism leading to the formation of modules. Fundamentally, this rewiring mechanism is based on topological overlap (TO) (Ravasz, Somera, Mongru, Oltvai, & Barabási, 2002). The origin of the TO concept stems from applications of set theory to nodes graph in network analysis, which became established as a relevant approach for quantifying the similarity of nodes in terms of their common network neighborhoods; for a review focusing on bipartite graphs see Bass et al. (2013). TO is closely related to the matching index (Hilgetag, 1999; Hilgetag et al., 2000; see also Hilgetag, Kötter, Stephan, & Sporns, 2002; Sporns, 2003), an adaptation of the Jaccard index to neighborhoods of nodes in a graph. Higher-order variants of this quantity have also been discussed in the literature (Li & Horvath, 2006).

Prompted by the exploration of network motifs (that is, few-node subgraphs which are often statistically enriched in real networks (see Milo et al., 2004, 2002), the interplay of different topological scales in a graph has become an object of intense research. In particular, several studies have shown that global network properties, such as hierarchical organization (Vazquez et al., 2004) or modularity (Fretter, Müller-Hannemann, & Hütt, 2012), can systematically affect the composition of networks in terms of local topology or network motifs (see also Reichardt, Alamino, & Saad, 2011). Intriguingly, that line of research inspires the complementary possibility: a systematic iterative selection on local network structures may conversely install, or at least enhance, certain global network properties. This is the conceptual approach we set out to explore here, where our topological reinforcement rule iteratively enhances the local topological overlap.

As a further step, we explore a plausible dynamical implementation of the topological reinforcement. We use an excitable network model, the SER model, in which the discrete activity of network nodes is described by susceptible, excited, and refractory states, representing a stylized neuron or neural population. In this case, the plasticity acts in a Hebbian-like fashion based on the functional connectivity (FC) derived from co-activation patterns of network nodes. The results confirm a correspondence between the two plasticity modalities, which speaks in favor of the dynamical implementation representing a biologically plausible mechanism through which topological reinforcement may take place in real systems, thus supporting the role of topological reinforcement as a contributor to the emergence of modular brain networks.

## RESULTS

Starting from initial random configurations, we evolved networks according to the topological reinforcement rule. Topological reinforcement was based on the TO between nodes of a network. At each rewiring step, a randomly selected node was connected to a non-neighbor with the highest TO, while pruning another link with random uniform probability, in order to preserve network density.

### Random Networks Evolve Towards Modular, Small-World Organization

**Figure 1.**
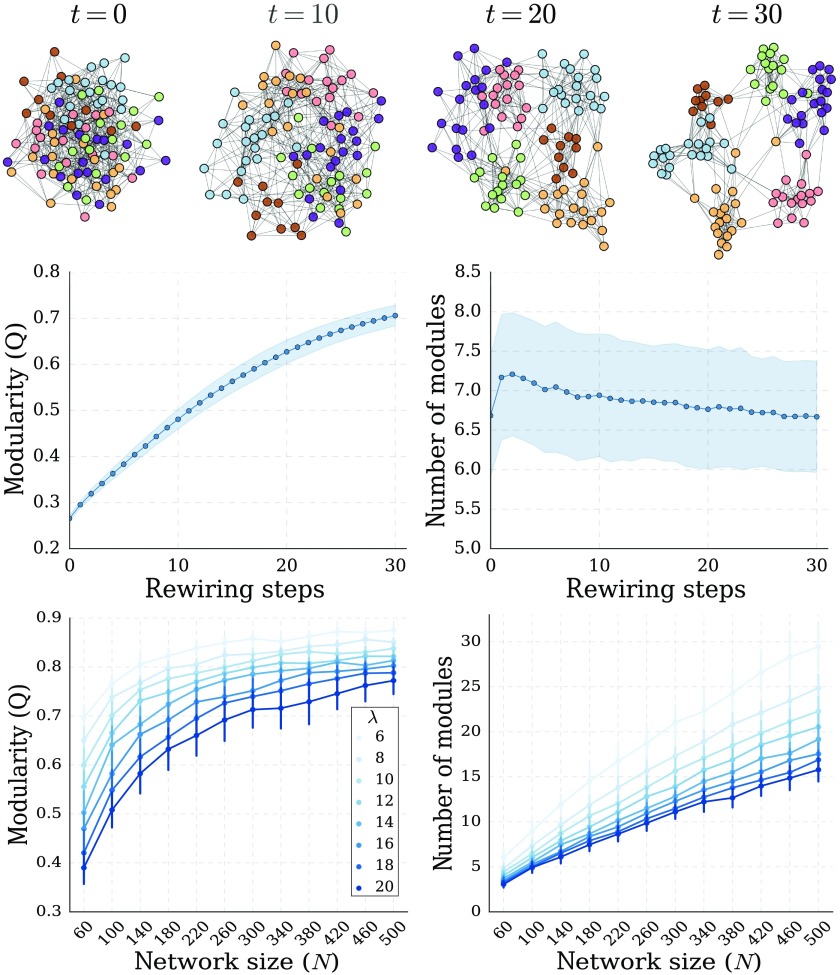
Emergence of modular network organization from topological reinforcement. (Top) Example of network evolution resulting from topological reinforcement, starting from a random network. Layouts are generated according to the Fruchterman-Reingold force-directed algorithm. Nodes are consistently colored according to the final modular structure. At each rewiring step (*t*), a total of *N* links where reallocated. (Middle) Evolution of the modularity (Q) and number of modules as a function of the number of rewiring steps (mean and standard deviation across 500 simulation runs). (Bottom) Final modularity (left) and number of modules detected (right) for different network sizes (*N*) and densities (*λ*, average number of links per node) (mean and standard deviation across 50 independent graph realizations).

The topological reinforcement rule reliably evolved synthetic random networks toward high modularity (Figure 1). Moreover, due to increased clustering, the final networks had a small-world organization (Supporting Information Figure S1, Damicelli, Hilgetag, Hütt, & Messé, 2019). The results were robust across multiple runs and multiple initial network realizations (Supporting Information Figure S2, Damicelli et al., 2019). We also explored the effect of network size and density on the outcome of the TR rule (Figure 1). The results were consistent, showing similar scaling curves across conditions, which speaks for the robustness of TR in generating modular networks.

The scaling pattern of the final number of modules could be roughly approximated based on the average network degree. The rationale is that the number of modules is proportional to the number of nodes while inversely proportional to the average size of neighborhoods containing nearest and next to nearest neighbors. As we did not have an analytical expression for the sizes of such neighborhoods, we assumed that it is proportional to *λ*^1 +*a*^, where *a* is some exponent with *a* < 1. The exponent 1 accounts for nearest neighbors and *a* for the double counting of nodes when going to next-to-nearest neighbors. We observe a good (though not perfect) agreement with the numerical results for $a\approx \frac{1}{4}$ (see Supporting Information Figure S3, Damicelli et al., 2019).

### Final Network Structure Reflects Initial Network Organization

**Figure 2.**
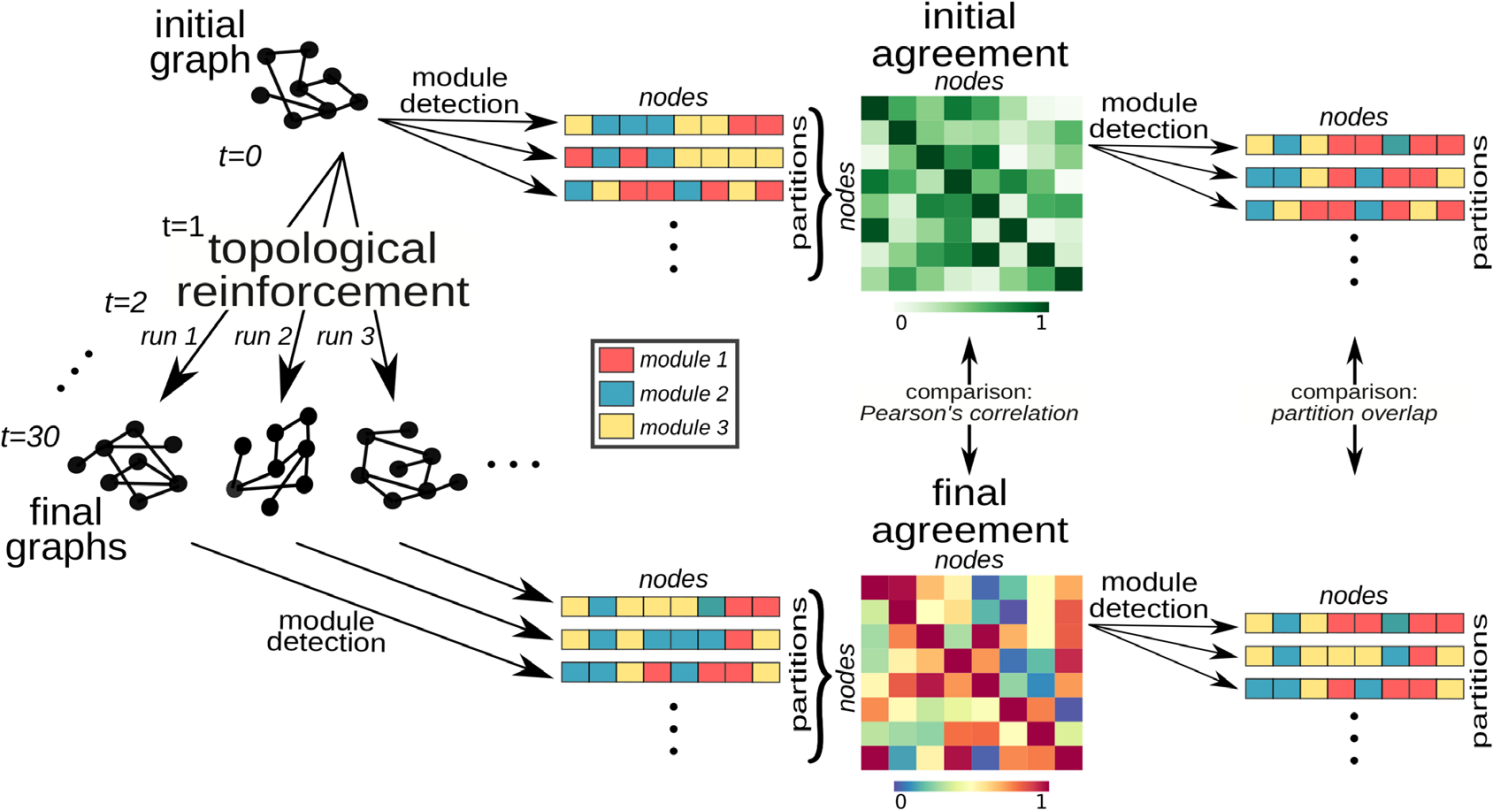
Module agreement and “proto-modules.” Schematic example for a graph with *N* = 8 nodes and 30 rewiring steps of the procedure for probing the existence of “proto-modules” in the initial graph and the relationship between initial and final network structure. Each simulation run starting from the same initial graph is represented by a grey color. A schematic representation of the affiliation vectors can be viewed under *module partitions*. Each element of the vector represents a node and its color indicates the module that it was assigned to. The probability that two nodes end up in the same module across partitions is represented by the *agreement* matrix, in other words, a consensus across module partitions. The agreement matrices where compared both in terms of their values (Pearson’s correlation) and their modular composition (partition overlap). See Methods for details.

The topological reinforcement rule amplified weak “proto-modules” already present in the initial random graph. The similarities between the initial and final network structures were investigated in terms of Pearson correlation and partitions overlap between networks; see Methods section and Figure 2 for details. Module detection is an algorithm that results in the assignment of nodes to mutually exclusive groups, *modules*. The outcome may be deterministic or stochastic, depending on the specific algorithm. Module partition is a representation of the modules and their nodes as a so called *affiliation vector*. Module agreement matrix is also referred to as *consensus*, where each cell represents the frequency with which every two nodes were assigned to the same module. High values indicate that nodes where often classified in the same module.

**Figure 3.**
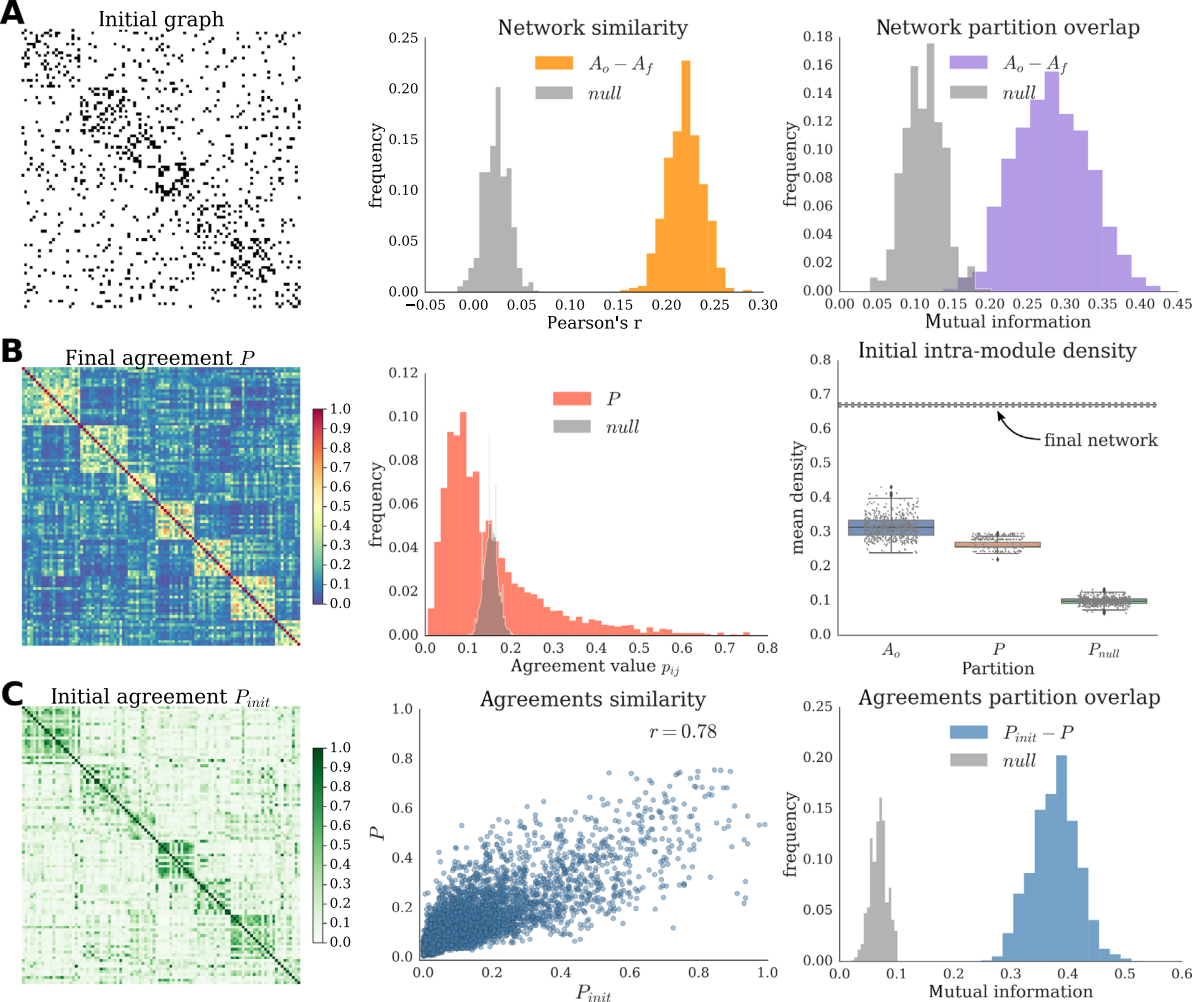
Relationship between initial and final network structures. (A) Initial adjacency matrix (left) reordered according to the modular partition of the agreement *P*. Similarity (middle) and partition overlap (right) between all pairs of initial and final networks, and the corresponding null distributions. (B) Agreement matrix across multiple runs (*P*, left) reordered according to its modular partition. Histogram of the *P* values and of the corresponding null model (middle). Distributions of the intramodular density of the initial network (right). Average intramodule density of the initial network according to different types of module partitions. The procedure was repeated 500 times for each type of partition. As a reference, the mean intramodule density of the final network modules is also plotted (average and standard deviation). (C) Initial agreement matrix (*P*_*init*_, left) reordered according to the modular partition of *P*. Similarity (middle) and partition overlap (right) between *P*_*init*_ and *P* and the corresponding null distribution.

Statistical analysis across multiple runs showed a significant similarity and partition overlap between the final graphs and the initial one (Figure 3A). Moreover, the results also showed a consistent pattern of final modular organization (Figure 3B). The module agreement of final networks across multiple runs (*P*) displayed pairs of nodes with high probability (beyond chance) to end up in the same module. Figure 3B shows the mean intramodule density of the initial random graph according to different partitions. The distribution of the mean intramodule density according to the modules detected in the agreement *P* coincided fairly well with the mean intramodule density of the partitions detected on the graph itself. In contrast, intramodule density from partitions coming from a null model was centered around 0.1, that is, the graph density (i.e., probing density of randomly chosen groups of nodes). In the random graphs used as initial condition, no variations in link density are expected (since, by definition, connection probability is uniform for all pairs of nodes). Importantly, that is the case *on average across graph realizations*, but, because of stochastic variations and finite-size effect, individual graphs might contain groups of nodes with slightly higher density of edges than expected. We refer to these groups as “proto-modules.” In order to highlight these modules, a module detection algorithm was applied multiple times on the initial graph, and a module agreement matrix was built (*P*_*init*_). The correspondence between the initial and final network structures was also evident comparing the final agreement *P* with its analogous on the initial graph *P*_*init*_ (Figure 3C). The similarity (as measured by correlation) between both agreements was high. Additionally, we generated a set of partitions from *P* and another set of partitions from *P*_*init*_, and quantified the overlap between all possible pairs of partitions *P*_*init*_-*P*. We observed a significant overlap between the partitions from *P*_*init*_ and those from *P*. Furthermore, the results were robust across multiple initial network realizations (Supporting Information Figure S4, Damicelli et al., 2019).

### Biological Implementation of Topological Reinforcement

In the brain, topological reinforcement may be implemented through various plausible activity-based models. We explored one such model, in which the activity of network nodes was described by discrete susceptible, excited, and refractory states, the SER model, representing a stylized neuron or neural population. TR when transposed into biological context simply corresponds to the so-called Hebbian rule, where we substituted FC for TO, see Methods section for details. In order to explore the FC-based rule and its relation to TR, we exploited an interesting feature of the SER model: for a given graph topology, the relationship between TO and FC varies according to the parameters of the model. More specifically, the SER model allows both deterministic and stochastic formulations, depending on the definition of the state transition probabilities. In the deterministic case, only the initial proportions (*e*, *s*, *r*) of nodes in each state may vary, since the stochastic transition probabilities are fixed (*f* = 0 and *p* = 1). Whereas in the stochastic case, different parameter constellations may be achieved by varying such state transition probabilities (for more details, refer to Methods and Messé et al., 2018).

#### Rewiring rules comparison.

**Figure 4.**
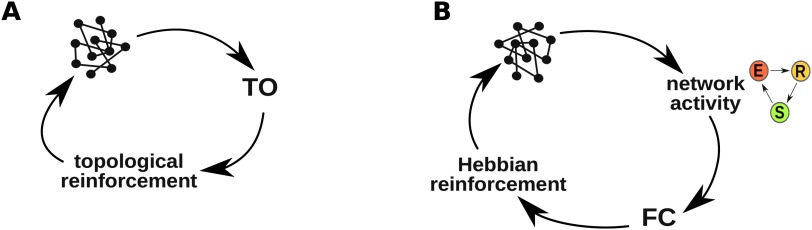
Rewiring schemes. (A) Topological Reinforcement. (B) Biological implementation - Hebbian rule.

We applied two different model scenarios (Figure 4). The first one, based solely on the topology, and we applied the topological reinforcement (TR) rule, which is based on the topological overlap (TO). While the second considered activity on the nodes (SER model), and the rewiring occurred in a Hebbian fashion, that is, based on functional connectivity (FC) between nodes and reinforcing connections between highly correlated nodes. The following schemes show the core loops of both schemes for comparison. Each iteration of a loop is equivalently denoted as a rewiring step. See Methods for more details.

**Figure 5.**
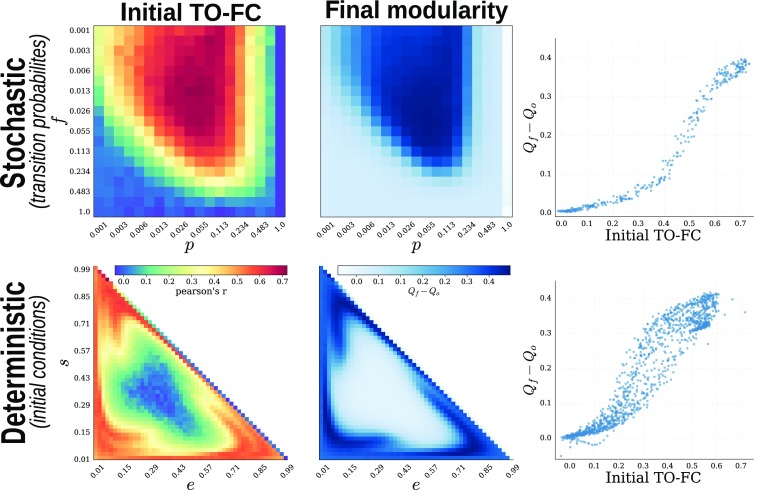
Biological implementation of the topological reinforcement. Parameter space exploration of the stochastic (top) and deterministic (bottom) SER model. Similarity (measured by correlation) between TO and FC in the initial graph (left), final modularity (middle) expressed as the difference between the mean final modularity value and the modularity of the initial random graph (across multiple (500) community detection). (Right) Scatter plot of the relationship between both quantities. Note logarithmic scale for the stochastic case.

**Figure 6.**
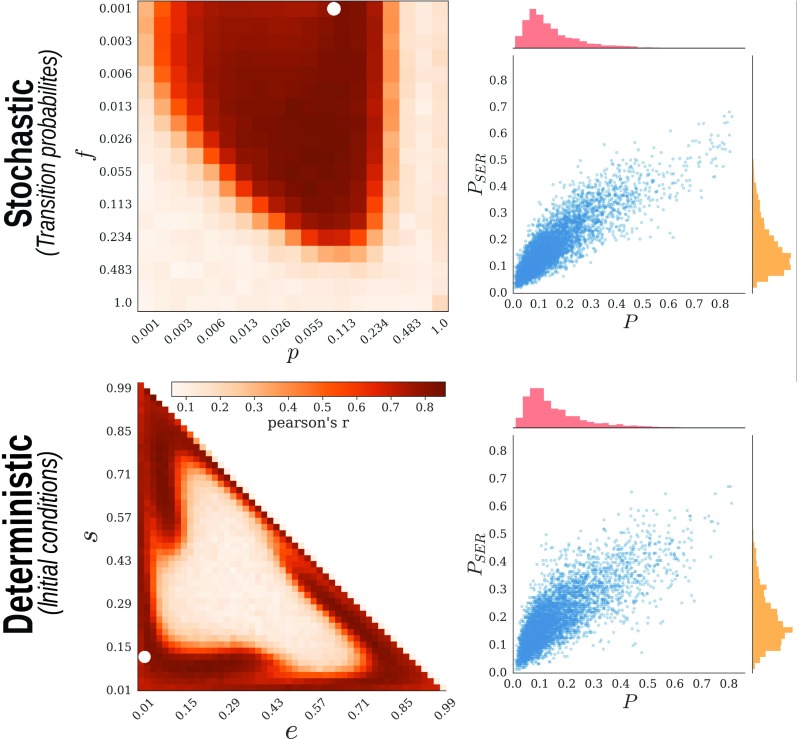
Correspondence between the topological reinforcement and the Hebbian rule. Similarity between *P* from the topological reinforcement and from the Hebbian rule using the stochastic (top) and deterministic (bottom) SER models. Pearson’s correlation coefficient was computed to summarize the similarity between both rules across the parameter spaces. Scatter plots represent the relationship for a selected setting (white dots in the heat-maps).

After exhaustive evaluation of the possible constellations for each case, we found the following: first, that the FC-based rule was also able to generate a modular network structure. Importantly, a sufficiently high similarity (as measured by correlation) between TO and FC within the initial configuration was a necessary condition for modularity emergence, as illustrated by the sharp transition from the nonmodular to the modular regime (Figure 5); second, the results produced by the FC-based plasticity were consistent with the ones from TR, both in terms of final network configurations and their module partitions (Figure 6). Fundamentally, this indicates that, provided the correlation between TO and FC is high enough, the Hebbian rule acts indirectly as topological reinforcement.

## DISCUSSION

The importance of segregation in the brain is supported by numerous studies (Sporns & Betzel, 2016; Wig, 2017). However, there is a lack of general mechanisms explaining the emergence of brain modularity. In the present study, we propose an explicit mechanism of reshaping local neighborhoods through topological reinforcement that might act as a fundamental principle contributing to the emergence of modules in brain networks. In addition, our work shows that a Hebbian rule acting on an activity-based model may actually be instantiating the same underlying rewiring pattern responsible for the modules creation, that is, the topological reinforcement.

Given accumulated evidence that global network properties can systematically affect the composition of local network structures, such as motifs (Fretter et al., 2012; Reichardt et al., 2011; Vazquez et al., 2004), we propose a complementary bottom-up approach that is acting locally in order to shape global features. Our proposed mechanism is in line with empirical data where “homophily” appears as an essential feature of brain connectivity. At the micro scale, it has been shown that the probability of finding a connection between a pair of neurons is proportional to their number of shared neighbors (Perin, Berger, & Markram, 2011) whereas at the macro scale, the strength of connections between brain regions tends to be the higher the more similar their connectivity profiles are (Goulas, Schaefer, & Margulies, 2015).

Our results show that topological reinforcement reliably and robustly produces modular network architectures over time, accompanied by the small-world property. Additionally, the final modular organization of the networks seems to correspond to groups of nodes in the initial networks that have higher than average connection density. As such, our rewiring mechanism acts as an amplification of these “proto-modules,” similarly to a previously reported effect in weak modular weighted networks evolving under a Hebbian rule based on chaotic maps synchronization (Yuan & Zhou, 2011).

We extended the framework of topological reinforcement by introducing a plausible biological implementation. Our dynamical model choice, the SER model, offers the advantage of capturing essential characteristics of stylized neuronal activity while being more tractable than detailed typical models. This minimalistic excitable network model has a rich history across disciplines and in particular in neuroscience (Anderson & May, 1992; Bak, Chen, & Tang, 1990; Drossel & Schwabl, 1992; Furtado & Copelli, 2006; Kinouchi & Copelli, 2006), where it can capture nontrivial statistical features of brain activity patterns (Haimovici, Tagliazucchi, Balenzuela, & Chialvo, 2013; Messé, Hütt, König, & Hilgetag, 2015). This model has also been used to study the impact of network topology, such as modules, hubs, and cycles, on network activity patterns (Garcia, Lesne, Hilgetag, & Hütt, 2014; Messé et al., 2015; Müller-Linow et al., 2008). A relative-threshold variant (requiring a certain percentage of a node’s neighbors to be active, in order to activate the node) was explored in Hütt, Jain, Hilgetag, and Lesne (2012) and Fretter, Lesne, Hilgetag, and Hütt (2017). The deterministic limit of the model (*p* → 1, *f* → 0) has been analyzed in Garcia, Lesne, Hütt, and Hilgetag (2012) and in much detail in (Messé et al., 2018).

In the biological implementation, the topological reinforcement rule was reformulated by using functional connectivity (FC) as a surrogate of TO. These results were consistent with TR, indicating that the biological implementation acted indirectly at the topological level. In other words, the FC served as a proxy of TO, and therefore Hebbian reinforcement led indirectly and ultimately to the topological reinforcement of a modular network organization. The explanation for this finding is based on the fact that, for suitable dynamical regimes and structural architectures, FC is positively correlated with TO in excitable networks (Messé et al., 2018), which is intuitive if one considers that common inputs may promote correlations. Thus, we propose the topological reinforcement principle as an underlying common ground, bridging an activity-based Hebbian model and a purely topological generative model.

Our results are in line with recent theoretical work on the contribution of specific network motifs to higher-order network organization, in which the reinforcement of connections between neurons receiving common inputs led to the formation of self-connected assemblies (Ravid Tannenbaum & Burak, 2016). Hence, our Hebbian plasticity scenario exploited the correspondence between TO and FC as it could be observed with the exploration of different SER parameter constellations. These parameters promote different relations between TO and FC, and we found that such a dependence systematically predicted the emergence (or not) of modular networks.

Previous computational studies have shown that evolutionary algorithms of network connectivity optimizing, for example, functional complexity (defined as balance between segregation and integration) can lead to modular network formation (Sporns, Tononi, & Edelman, 2000). Such findings point to the relevance of modularity as a crucial organization principle underlying complex functional brain processes. Nevertheless, these models do not provide a biologically interpretable and implementable mechanism, since the explicit global optimization function (functional complexity) cannot be directly interpreted as a biological mechanism shaping brain connectivity.

In the sense of biological plausibility, activity-based plasticity models (e.g., based on Hebbian plasticity) constitute a more directly interpretable approach. Previous studies have used a variety of neural activity models ranging from abstract representations, such as chaotic maps (van den Berg & van Leeuwen, 2004) and phase oscillators (Gleiser & Zanette, 2006), to more physiologically realistic models, such as neural masses (Stam, Hillebrand, Wang, & Van Mieghem, 2010) and spiking neuron (Kwok, Jurica, Raffone, & van Leeuwen, 2006) models. In general, Hebbian reinforcement led to the formation of modular architectures, consistent with our results for the excitable model. Interestingly, as a practical biological example beyond the pure theoretical realm, this type of plasticity-guided modular emergence has recently been studied also in real neural activity in zebrafish larvae (Triplett, Avitan, & Goodhill, 2018), pointing to the relevance of the current results. The open question for this type of models concerns the specific underlying topological changes that they promote, since these studies focus on the implementation of the phenomenon (based on the activity) and not on the algorithmic level (the topological dimension) and both levels interact in nontrivial ways. Indeed, some of these models even showed that final topological features (e.g., number of modules) might purely depend on properties of the dynamical model (Yuan & Zhou, 2011). In other words, they did not provide insights about a general mechanism specifying which topological changes might be necessary for the emergence of modular structure. Compared with this group of models, our model is different in that the topological reinforcement principle is agnostic with respect to the specific dynamical regime, and it explicitly addresses the topological changes that take place in the network.

An alternative modeling approach is provided by generative models, where typically a given probability function governs the insertion of links and/or nodes during simulations (Betzel & Bassett, 2017). Recent work has shown that including homophily as a factor to determine connection probability (and after proper data-driven parameter tuning) makes it possible to account for a great deal of functional (Vértes et al., 2012) as well as structural (Betzel et al., 2016) topological features of real large-scale brain networks. Although these studies provide a valuable basis for confirming the importance of TO as an essential feature and reducing the dimensionality of brain connectivity to a few model parameters (Betzel & Bassett, 2017), disentangling the mechanistic nature of the phenomena (e.g., modularity emergence) turns out to be nontrivial, since information about the final state might be explicitly built-in in the generative model. But even more crucially, how the generative function is actually implemented in real systems is out of the scope of this kind of modeling approach. As a complement to this group of models, our contribution offers a concrete scenario in which a generative mechanism can actually be implemented in a biologically more realistic fashion.

In summary, as expected for any modeling approach, a trade-off exists between generative and activity-based models. Phenomenological descriptions and mechanistic explanations complement each other and a gap remains for explaining how they link to each other. Our contribution represents an attempt to address this gap: first, by providing an explicit topological mechanism of module formation (generative mechanism); second, by trying to reconcile such an abstract level of analysis with the biological implementation, by means of an activity-based formulation of the model.

The present results are subject to several methodological considerations. For example, our study did not take into account a geometrical embedding and rather focused on the pure topological contribution of the topological reinforcement. Although we recognize that the brain is a spatially embedded system and that physical constraints, such as wiring-cost, play a fundamental role shaping brain connectivity (Henderson & Robinson, 2013), previous studies have shown that, in addition to them, topological aspects are essential to describe real connectomes (Betzel et al., 2016; Kaiser & Hilgetag, 2006). Thus, we aimed at isolating the topological effect and avoiding the situation in which geometric constraints, such as the distance-dependent probability of connection used in previous studies (Jarman, Trengove, Steur, Tyukin, & van Leeuwen, 2014), introduce already by themselves a clustered connectivity, thus potentially overriding the changes based on the topology itself. Specifically for the case of our model, an initial spatially constrained, distance-dependent connectivity could also create “proto-modules” on which the connectivity would develop.

For sufficiently long simulations, a stationary behavior is observed. However, because of their relative simplicity, the rules tend to disconnect the evolving networks (see Supporting Information Figure S5, Damicelli et al., 2019). This consequence can also be found in previous studies with this type of models, where other modeling choices were made, such as discarding runs with disconnections or explicitly using network size and density that avoid such a scenario (Rubinov, Sporns, van Leeuwen, & Breakspear, 2009; van den Berg & van Leeuwen, 2004). From a practical point of view, we chose a number of rewiring steps that avoids such scenario. We recognize an interesting line for future work taking into account possible counteracting mechanisms that might balance out disconnections and add realism to the model.

Other interesting potential variations of the presented model for future work could include networks with weighted edges where the plasticity rule acts regulating the weights, as well as model settings simulating developmental pruning processes, where the total network density decays over time.

Regarding the plausible biological implementation, we chose a simple abstract model for computational tractability. It would be interesting to compare our framework with more biologically realistic dynamical models, such as networks of spiking neurons.

## CONCLUSIONS

Our findings suggest a selective reinforcement of the topological overlap as a plausible mechanism contributing to the modular organization of brain networks. Moreover, under appropriate conditions, functional connectivity might act as a proxy, or a dynamical representation, of topological overlap. Thus, biological-inspired plasticity rules, such as the Hebbian rule, can indirectly promote modularity. To our knowledge, these findings constitute a first topologically mechanistic explanation of module formation in complex brain networks and its link to a physiologically plausible realization. Despite the simplicity of our framework, we trust it to carry a conceptual value that contributes to the long challenging path of understanding the fundamental principles of brain organization.

## METHODS

### Networks

We considered synthetic undirected networks without self-connections of size *N* = 100 nodes and average connectivity *λ* = 10 (equivalently, a density of 0.1). The networks were represented by a symmetric adjacency matrix A, where *a*_*ij*_ = 1, if nodes *i* and *j* are connected, 0 otherwise. Initial networks were generated according to the classical Erdős-Rényi model (Erdős & Rényi, 1959).

We explored the robustness of the plasticity rule across various network realizations and multiple runs (using the same initial network). We generated 100 synthetic random initial graphs and performed 500 runs for each of them. In order to study the scaling properties of our model, we also evaluated graphs with different densities (*λ*, average number of links per node, ranging between 6 and 20 by step of 2) and size (*N*, varying between 60 and 500 by step of 40).

### Topological Reinforcement

Topological reinforcement was based on the topological overlap metric. TO represents the neighborhoods similarity of a pair of nodes by counting their number of common neighbors (Ravasz et al., 2002):

$$to_{ij}=\frac{\sum_{k}a_{ik}a_{kj}+a_{ij}}{\text{min}(\sum_{k}a_{ik},\sum_{k}a_{kj})+1-a_{ij}}.$$ (1)

At each rewiring step, the rule connected a randomly selected node that is neither disconnected nor fully connected with a nonneighbor with the highest TO, while pruning another link with uniform probability, hence preserving graph density. For computational efficiency, the rewiring was applied by inserting simultaneously one link on $\frac{N}{2}$ random different nodes at each step, and pruning the same number of links at random, so that $2\frac{N}{2}=N$ links were reallocated at each rewiring step, with statistically equivalent results as when only two links (one insertion, one pruning) per step were modified. In order to compare the results across different graph sizes and densities, we computed the length of each run, *r*, by fixing the average number of rewiring per link, *K*, so that $r=\frac{\lambda NK}{2N/2}=\lambda K$. Throughout the manuscript *K* = 3, which ensures that the networks remain connected (see Supporting Information Figure S5, Damicelli et al., 2019 for details).

### Excitable Model

We used a three-state cellular automaton model of excitable dynamics, the SER model. The activity evolves according to the following synchronous transition rules:

▪  S → E, if at least one neighbor is excited; or with probability *f* (spontaneous activation);
▪  E → R;
▪  R → S, with probability *p* (recovery).

In the deterministic SER scenario, that is, *f* = 0 and *p* = 1, for each network and initial condition setting, the activity time windows consisted of 5,000 runs of 30 time steps each and FC was averaged over runs. The initial conditions were randomly generated, covering the full space of possible proportions of states. In the stochastic SER scenario, that is, *f* > 0 and *p* < 1, for each parameter setting (*f*,*p*), the activity time window consisted of one run of 50,000 time steps. The initial conditions were randomly generated with a proportion of 0.1 nodes excited, while the remaining nodes were equipartitioned into susceptible and refractory states.

### Functional Connectivity

To analyze the pattern of excitations in the SER model, we computed the number of joint excitations for all possible pairs of nodes. The outcome matrix is the so-called co-activation matrix, a representation of the functional connectivity of the nodes is as follows:

$$c_{ij}=\underset{t}{\sum }1_{E}(x_{i}^{t})1_{E}(x_{j}^{t}),$$ (2)

where $x_{i}^{t}\in S,E,R$ being the state of node *i* at time *t*, and 𝟙_*E*_ the indicator function of state E. FC was then normalized to scale values between 0 and 1:

$$fc_{ij}=\frac{c_{ij}}{\min(c_{ii},c_{jj})}.$$ (3)

### Biological Implementation: Hebbian Rule

When transposing the topological reinforcement into a biological context, by using a plausible model of brain dynamics, it turns out that the rule corresponded to the well-known Hebbian rule, in which we substituted FC for TO. In other words, the rewiring events occurred with the exact same algorithm, but based on the FC derived from the activity during the given time window. Thus, we used the SER model for activity simulation during a time window after which FC was derived and the rewiring was applied: a random node was selected and connected to a nonneighbor node with maximum FC, while a link was selected randomly with uniform probability and pruned. Once rewired, we iterated through the same steps until the end of the simulation. As for the topological reinforcement and for computational efficiency, the rewiring was applied simultaneously on $\frac{N}{2}$ different nodes at each step. In order to keep the final networks comparable, the total number of rewiring steps was the same for both plasticity modalities, as defined above. According to the SER scenario, stochastic or deterministic, we evaluated the model for different parameter constellations or initial conditions, respectively. For one initial graph, we studied each possible combination of parameter constellation/initial condition by performing 150 simulation runs, and the final graph measures were averaged across runs.

### Network Analysis

Synthetic graph realizations, basic graph properties (clustering, path length, small-world), community detection, matrix reordering, and graph layouts were performed using the Brain Connectivity Toolbox (Rubinov & Sporns, 2010) (Python version 0.5.0; github.com/aestrivex/bctpy) and NetworkX (Hagberg, Schult, & Swart, 2008). For a given graph, communities were extracted by means of the Louvain algorithm that attempts to maximize the modularity of the network by using the so-called Q value (Blondel, Guillaume, Lambiotte, & Lefebvre, 2008). Similarity between networks and agreements was assessed by means of the Pearson correlation between their connectivity matrices. Overlap between partitions was probed based on the normalized mutual information between the communities (Meil, 2007).

### Module Agreement and “Proto-Modules”

From a given initial network, multiple simulation runs (500) were performed, and the community detection algorithm was applied on each final graph to find a partition of the nodes into communities. Then, an agreement matrix *P* was computed across all final partitions, where *p*_*ij*_ quantifies the relative frequency with which nodes *i* and *j* belonged to the same community across partitions. Finally, the community detection algorithm was applied 100 times on *P*, yielding a representative set of final partitions of the nodes into non-overlapping communities given an initial graph (Figure 2). In order to probe the structure of each initial graph and find potential “proto-modules,” we applied the community detection on the initial graph. Because of the weak signal of random graphs, the stochasticity and associated degeneracy of classical community detection algorithms, a consensus clustering was employed to generate stable solutions. For each random initial graph, the community detection algorithm was applied 500 times, then an agreement matrix was computed, named *P*_*init*_, and finally the community detection algorithm was applied 100 times on this agreement matrix, yielding a representative set of (stable) partitions of the initial graph (Figure 2).

### Statistical Assessments

In order to assess the significance of the results, null network models were generated. When comparing networks in terms of similarity (by Pearson correlation), a null model was generated by randomly rewiring a given graph (once per link), while preserving the degree distribution (Maslov & Sneppen, 2002). Two null models where used when comparing networks in terms of partition overlap. For comparison of individual runs (initial vs. final structures or initial vs. final agreements), we simply used a rewired initial graph as explained above instead of the actual one that was used as initial condition for the run. As null model for the comparison of agreement matrices, a null agreement *P*_*null*_ was constructed by first shuffling the individual partitions (i.e., conserving the number of modules and their sizes, but randomly altering the nodes affiliation) and then computing the agreement across them. Thus, such a null model generated the expected distribution of agreement values that would occur purely by chance for a given number of nodes and modules of given sizes.

## AUTHOR CONTRIBUTIONS

Fabrizio Damicelli: Conceptualization; Investigation; Methodology; Software; Visualization; Writing – Original Draft; Writing – Review & Editing. Claus C. Hilgetag: Conceptualization; Funding Acquisition; Investigation; Project Administration; Supervision; Writing – Original Draft; Writing – Review & Editing. Marc-Thorsten Hütt: Conceptualization; Investigation; Methodology; Supervision; Validation; Writing – Original Draft; Writing – Review & Editing. Arnaud Messé: Conceptualization; Investigation; Methodology; Validation; Writing – Original Draft; Writing – Review & Editing.

## FUNDING INFORMATION

Fabrizio Damicelli, Deutscher Akademischer Austauschdienst (http://dx.doi.org/10.13039/501100001655). Claus C. Hilgetag, Deutsche Forschungsgemeinschaft (http://dx.doi.org/10.13039/501100001659), Award Id: HI 1286/5-1. Claus C. Hilgetag, Deutsche Forschungsgemeinschaft (http://dx.doi.org/10.13039/501100001659), Award Id: SFB 936/A1, Z3. Claus C. Hilgetag, Deutsche Forschungsgemeinschaft (http://dx.doi.org/10.13039/501100001659), Award Id: TRR 169/A2. Marc-Thorsten Hütt, Deutsche Forschungsgemeinschaft (http://dx.doi.org/10.13039/501100001659), Award Id: HU 937/7. Arnaud Messé, Deutsche Forschungsgemeinschaft (http://dx.doi.org/10.13039/501100001659), Award Id: SFB 936/Z3.

## TECHNICAL TERMS

Modularity: The existence of groups of nodes, referred to as communities or modules, that are more interconnected than with other nodes of the network. It can be quantified with an index called the Q value.
Topological overlap (TO): A metric quantifying the number of direct common neighbors between pairs of nodes.
Module detection: Algorithm that results in the assignment of nodes to mutually exclusive groups, that is, modules. The outcome may be deterministic or stochastic, depending on the specific algorithm.
Module partition: Representation of the modules and their nodes as a so called affiliation vector.
Module agreement matrix: Also referred to as consensus, where each entry represents the frequency with which every two nodes were assigned to the same module. High values indicate that nodes where often classified in the same module.
