# Supplementary material for: Topological reinforcement as a principle of modularity emergence in brain networks
Source: Netw Neurosci. 2019 May 1;3(2):589–605. doi: 10.1162/netn_a_00085 (PMC6542620; doi:10.1162/netn_a_00085)
Supplement: Supplementary file 1 [file netn-03-589-s001.pdf]

Damicelli, F., Hilgetag, C. C., Hütt, M., & Messé, A. (2019). Supporting information for "Topological reinforcement as a principle of modularity emergence in brain networks." *Network Neuroscience*, *3*(2), 589-605. https://doi.org/10.1162/netn_a_00085

Supportive Information

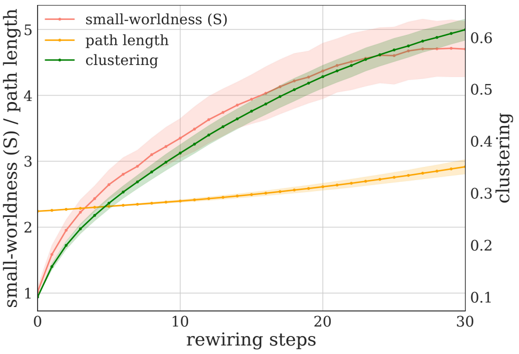

S1 Fig. Evolution of the mean clustering coefficient, characteristic path length and small-world index (S). Results are expressed as mean and standard deviation across 500 simulation runs as a function of the number of rewiring steps.

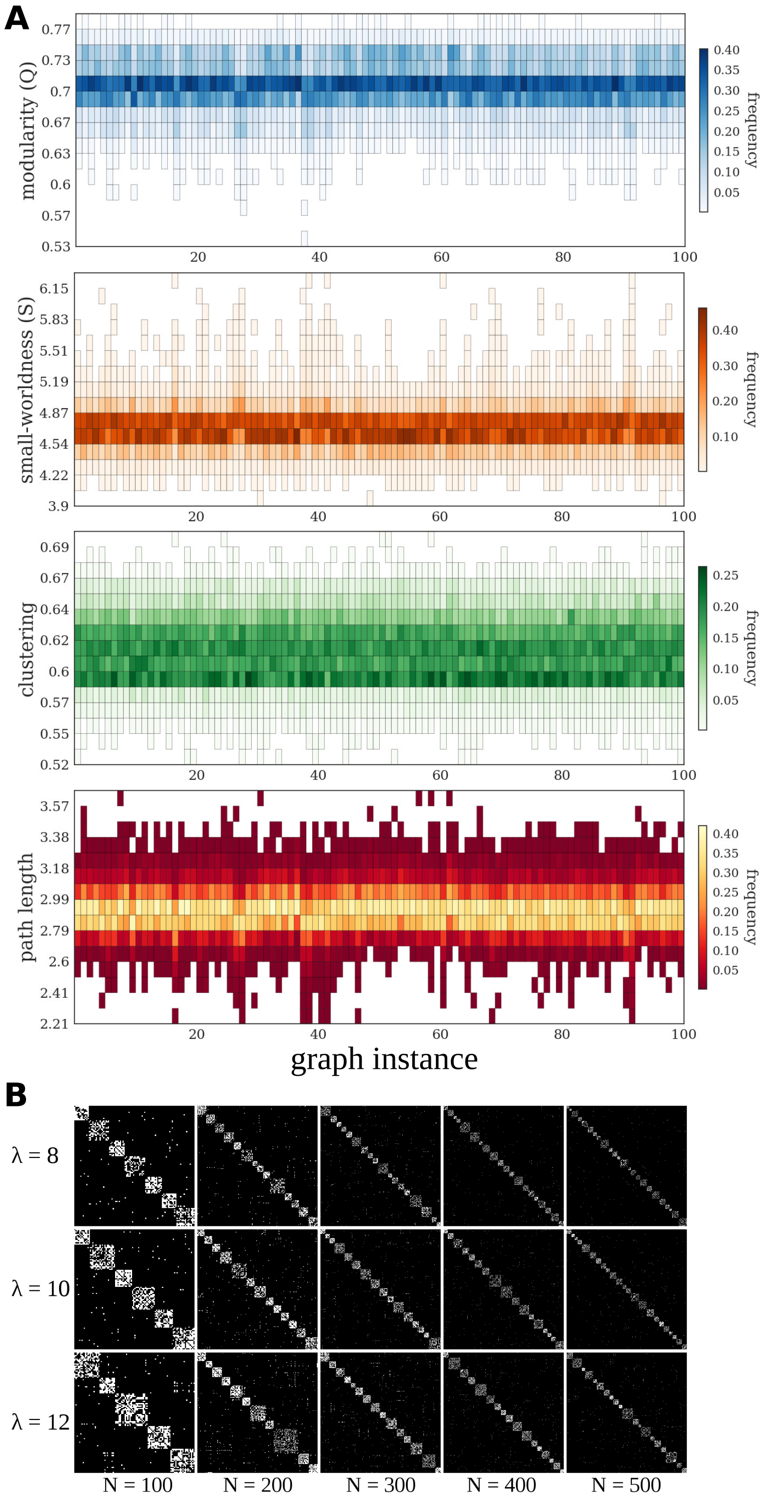

S2 Fig. Robustness of results for different initial random graph instances, final networks characteristics. (A) The heat maps show a summary of the results for 100 different initial random graph instances used as initial condition. For each one, 500 simulation runs were performed. For each final graph, modularity (Q), characteristic path length, mean clustering coefficient and and small-worldness coefficient (S) were computed. Each column of the heat map represents the distribution of values obtained across 500 simulations carried out with the same initial random graph instance. (B) Examples of final adjacency matrices of individual runs with different network sizes (N) and densities (λ).

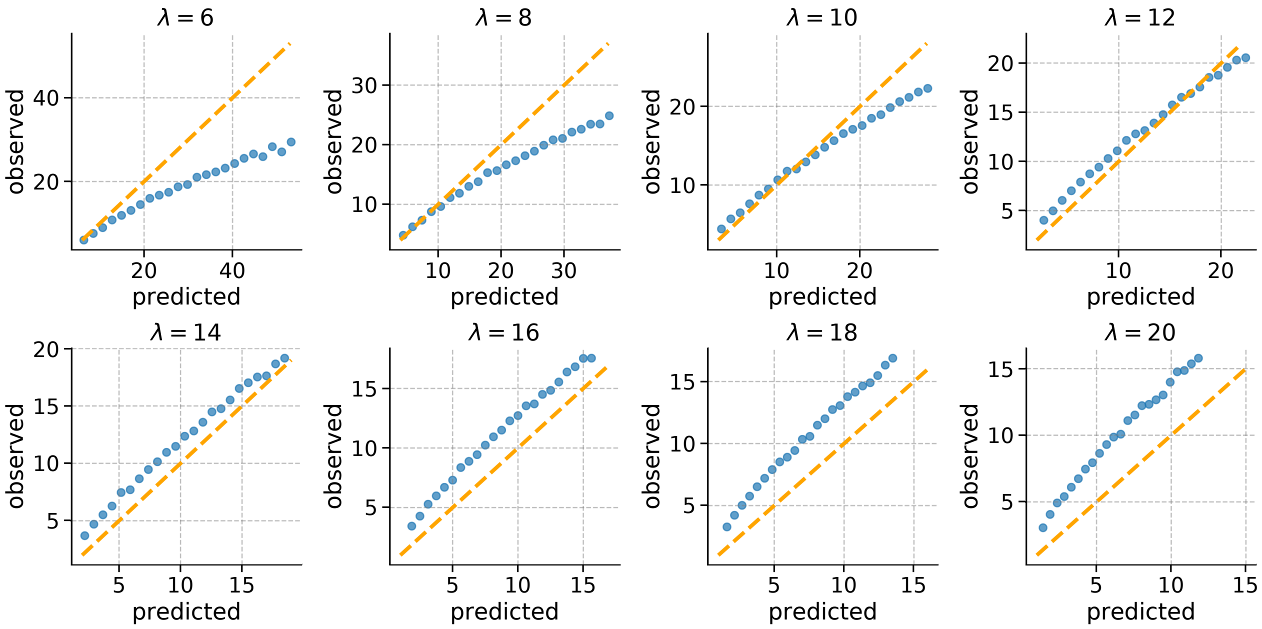

S3 Fig. Prediction of final number of modules. The scatter plots show the average number of observed modules across simulations versus its predicted values, estimated based on the average degree. Each panel represents an average network degree (6 to 20 by step of 2), and for each panel, each blue point corresponds to a network size (60 to 500 by step of 40). The orange dashed line shows the identity.

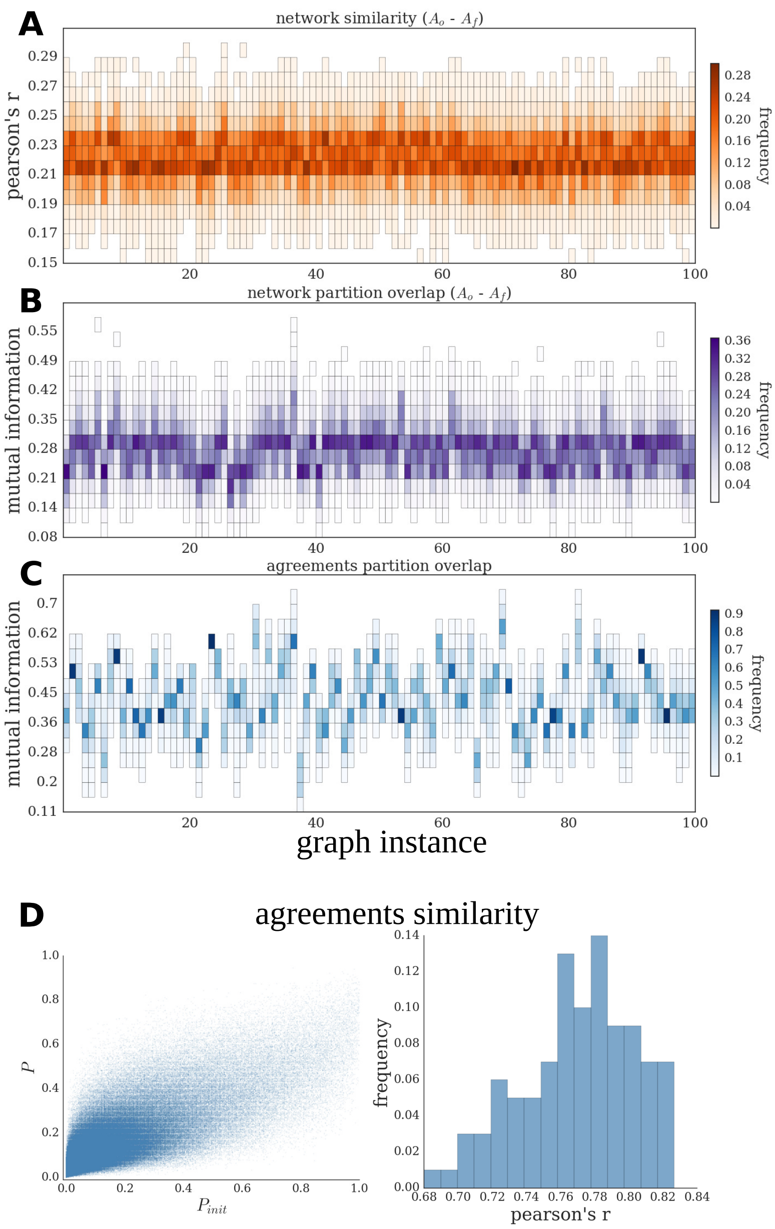

S4 Fig. Robustness of results for different initial random graph instances, correspondence between initial and final networks. (A-C) The heat maps show a summary of the results for 100 different initial random graph instances used as initial condition. Each column of the heat maps represents the distribution of values obtained across 500 simulations carried out with the same initial random graph instance. (A) Distribution of the correlation values between all pairs of initial and final networks. (B) Partition overlap between all pairs of initial and final networks (quantified as normalized mutual information, NMI). (C) Partition distance between initial (P_{init}) and final (P) agreements. (D) Similarity between P_{init} and P agreements, the scatter plot (left) shows the values over 100 different random graph instances used as initial condition, and the histogram (right) represents the Pearson's correlation coefficient between all pairs of P_{init}-P.

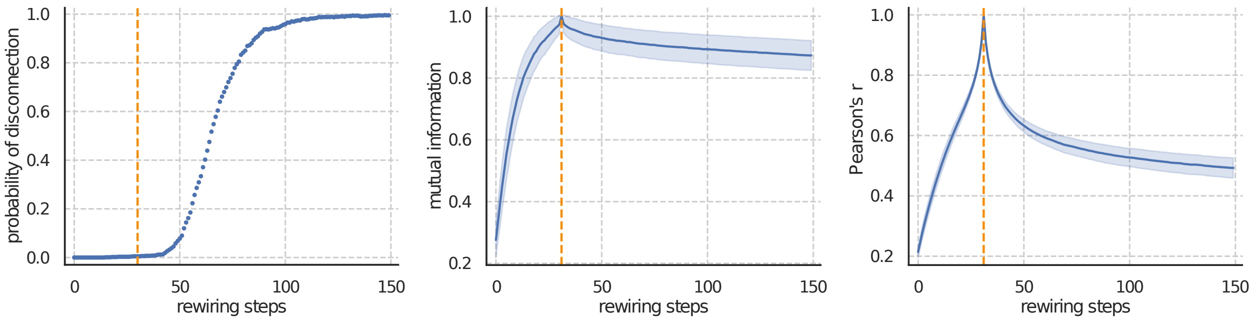

S5 Fig. Probability of network disconnection and steady state for long runs. (Left) Probability of network disconnection as a function of the number of rewiring steps. (Middle) Module partition overlap and (Right) correlation between the network at the typical final point (orange line) and at all other time points (shown as mean and standard deviation). In all cases, the orange line shows the typical length of our simulations. Results from 1000 runs (aggregated from 10 network instances, 100 runs each) of networks with $N = 100$ nodes and average connectivity $\lambda = 10$ (i.e., $density = 0.1$).
